# Comparison of Nasal Dimensions According to the Facial and Nasal Indices Using Cone-Beam Computed Tomography

**DOI:** 10.3390/jpm14040415

**Published:** 2024-04-14

**Authors:** Jeong-Hyun Lee, Hey-Suk Kim, Jong-Tae Park

**Affiliations:** 1 Department of Oral Anatomy, Dankook Institute for Future Science and Emerging Convergence, Dental College, Dan-Kook University, Cheonan 31116, Republic of Korea; 911105jh@dankook.ac.kr; 2 Department of Crime Scene Investigation Unit, Forensic Science Division, Daejeon Metropolitan Police, Daejeon 35403, Republic of Korea; kallaa@daum.net; 3 Department of Bio Health Convergency Open Sharing System, Dan-Kook University, Cheonan 31116, Republic of Korea

**Keywords:** nasal cavity, nasal index, facial index, CBCT, 3D modeling

## Abstract

The nasal cavity constitutes the foremost portion of the respiratory system, composed of the anterior nasal aperture, nostrils, and choanae. It has an intricate anatomical structure since it has various functions, such as heat exchange, humidification, and filtration. Accordingly, clinical symptoms related to the nose, such as nasal congestion, snoring, and nasal septal deviation, are closely linked to the complex anatomical structure of the nasal cavity. Thus, the nasal cavity stands as a paramount structure in both forensic and clinical contexts. The majority of relevant studies have performed comparisons between sexes, with studies making comparisons according to the FI and NI only and examining relative percentages. Furthermore, the nasal cavity was measured in 2D, and not 3D, in most cases. In this study, we conducted a 3D modeling and anthropometric assessment of the nasal cavity using a 3D analysis software. Furthermore, we aimed to investigate whether the size of the nasal cavity differs according to sex, facial index (FI), and nasal index (NI). We retrospectively reviewed the cone-beam computed tomography (CBCT) data of 100 participants (50 males, 50 females) aged 20–29 years who visited the dental hospital of Dankook University (IRB approval no. DKUDH IRB 2020-01-007). Our findings showed that nasal cavity sizes generally differed according to sex, FI, and NI. These findings provide implications for performing patient-tailored surgeries in clinical practice and conducting further research on the nasal cavity. Therefore, we believe that our study makes a significant contribution to the literature.

## 1. Introduction

The nasal cavity constitutes the foremost portion of the respiratory system, composed of the anterior nasal aperture, nostrils, and choanae [1]. It has an intricate anatomical structure since it has various functions, such as heat exchange, humidification, and filtration [2]. Accordingly, clinical symptoms related to the nose, such as nasal congestion, snoring, and nasal septal deviation, are closely linked to the complex anatomical structure of the nasal cavity [2]. Forensic science has recently been trending towards determining the sex of unidentified skeletal remains and performing facial reconstruction [3,4,5,6,7,8]. Sex determination, in particular, primarily relies on anatomical features, including the pelvis, mastoid processes, and nasal cavity size [9,10,11]. Among these structures, the nasal cavity has been reported to be a useful auxiliary tool for personal identification and age estimation [12,13,14]. Thus, the nasal cavity stands as a paramount structure in both forensic and clinical contexts. 

Cone-beam computed tomography (CBCT) technology is being used in many studies. Particularly, it allows for the observation of the complex craniofacial structure in 3D, enabling more precise analysis and diagnosis than traditional 2D methods [15]. As such, it is currently playing a central role in clinical research. Moreover, CBCT is now being utilized in the field of forensic science, unlike before. According to the research by Jayakrishnan [15], CBCT plays an important role in personal identification and facial reconstruction. Additionally, the study by Akbar et al. [16] conducted research on age estimation by measuring the dimensions and volume ratio of teeth using CBCT, and the studies by Azmi et al. [17] and Denny et al. [18] conducted gender analysis through the evaluation of the frontal sinus using CBCT. Thus, CBCT is being used as a tool necessary for identity verification in forensics, including age and gender. In light of this, this study also aims to conduct a analysis by modeling the nasal cavity in 3D, which is difficult to evaluate in 2D.

In contemporary society, facial contours have captured interest in foundational fields, such as anatomy and anthropology, as well as in clinical disciplines, such as plastic surgery. In forensic science, facial contours are essential structures for personal identification and sex determination [19]. Currently, there is ongoing research on the classifications of the facial index (FI) and nasal index (NI) [1,2,3,4,5,6,7,8,9,10,11,12,13,14,19,20,21,22]. FI classification differentiates facial shapes into hypereuryprosopic, euryprosopic, mesoprosopic, leptoprosopic, and hyperleptoprosopic based on facial length [20,21]. NI classification categorizes nose shapes into leptorrhine (long and narrow nose), mesorrhine (medium nose), and platyrrhine (broad nose) based on nasal length [22,23]. These facial and nasal shape classifications are being utilized not only in osteological assessments but also clinically [24,25,26].

Current research on the nasal cavity surpasses its forensic and clinical significance, also contributing to technological advancements in physical anthropology. The precision of measurements plays a critical role in advancing personal identification methodologies and enhancing our comprehension of human evolutionary processes. Consequently, this underscores the imperative need for research in three-dimensional technologies. Moreover, nasal cavity studies through 3D analysis enable a profound comprehension of nasal anatomy, offering the potential to significantly elevate the precision of clinical surgeries by addressing patient-specific anatomical discrepancies. This underscores the increasing importance of utilizing 3D technology in nasal research, necessitating further exploration in this field.

The majority of relevant studies have performed comparisons between sexes [1,2,3,4,5,6,7,8,9,10,11,12,13,14,19,20,21,22,23,24,25,26,27,28,29,30,31,32,33,34,35,36,37,38], with studies making comparisons according to the FI and NI only and examining relative percentages [1,2,3,4,5,6,7,8,9,10,11,12,13,14,19,20,21,22]. Furthermore, the nasal cavity was measured in 2D, and not 3D, in most cases [1,2,3,4,5,6,7,8,9,10,11,12,13,14,19,20,21,22,23,24,25,26,27,28,29,30,31,32,33,34,35,36,37,38]. However, the nasal cavity should be measured in 3D, as it consists of a roof, floor, medial wall, and lateral wall [1].

Mimics software is a program developed by Materialise, which is extensively utilized as an advanced medical imaging and 3D modeling tool in the medical industry. It easily converts medical scan data from CT and MRI scans into precise 3D models, enabling medical professionals to conduct more accurate diagnoses and create customized medical solutions for patients. The key utility of Mimics software lies in its ability to replicate accurate anatomical structures for patients, which is critically used for surgical planning, medical research, and educational purposes. For instance, surgeons utilize the 3D models provided by Mimics software to perform pre-surgical simulations, thereby preventing potential surgical risks and complications during actual operations. Furthermore, the software facilitates the development of customized medical devices and implants tailored to the unique anatomical structures of individual patients.

Mimics software provides a comprehensive suite of tools for the detailed analysis and measurement of anatomical structures. Users can utilize its advanced analytical tools to extract quantitative data, perform virtual surgeries, and evaluate procedural outcomes, offering a valuable resource for surgical planning and biomechanics research where understanding the interactions between different tissues and materials is essential.

Additionally, the software simplifies the complex process of 3D medical modeling, offering a user-friendly interface that makes it accessible for educational use. This ease of use allows students to employ the program effortlessly, enhancing their understanding of human biology and anatomical variations through hands-on experience with anatomical models.

In forensic medicine, Mimics is employed for personal identification purposes. It possesses applications for reconstructing unidentified remains, aiding forensic experts by modeling soft tissues over skeletal remains to deduce physical characteristics and potentially identify the deceased.

Overall, Mimics software provides essential tools for modern medical practices, including detailed anatomical visualization, custom implant design, surgical simulation, and educational applications. It represents a significant technological advancement in the medical field. To measure the complex structure of the nasal cavity, Mimics software was utilized.

In this context, this study aims to conduct a 3D modeling and anthropometric assessment of the nasal cavity using a 3D analysis software (Mimics, version 22.0, Materialise, Leuven, Belgium). Furthermore, we aim to investigate whether the size of the nasal cavity differs according to sex, FI, and NI. This study aims to provide data necessary not only for future medical approaches but also for significant implications from an anthropological perspective.

## 2. Materials and Methods

### 2.1. Study Population

The cone-beam computed tomography (CBCT) data of 100 participants (50 males, 50 females) aged 20–29 years who visited the dental hospital of Dankook University and had no tooth loss, asymmetry, or systemic diseases were obtained. The sample size was determined using the G-Power 3.1 (HHU, England) software. The data of patients whose treatments had already been completed were obtained for retrospective review; thus, an exemption from informed consent of this study was granted by the Institutional Review Board at Dankook University (IRB approval no. DKUDH IRB 2020-01-007).

### 2.2. Method

#### 2.2.1. CBCT Data

The CBCT images were taken by one technician. The participants were scanned in a manner to ensure the perpendicular alignment of the Frankfort horizontal plane to the ground to minimize distortions of the nasal cavity size across the participants. In addition, the CT scanner (Alphard 3030, Asahi, Kyoto, Japan) was positioned to match the midline of the face, and imaging was performed using specific settings: gantry angle at 0°, voltage set at 120 kV, and auto mA conditions. The following parameters were used for the CBCT: a slice increment of 0.39 mm, a slice thickness of 0.39 mm, a slice pitch set to 3, a scanning time of 4 s, and a matrix of 512 px × 512 px for image resolution. The resulting CBCT data were received in DICOM format. 

#### 2.2.2. Mimics 3D Modeling

The CBCT DICOM files were processed using Mimics (version 22.0, Materialise, Leuven, Belgium) to extract 3D patient data. The 3D modeling was performed in three views (coronal view, sagittal view, and frontal view) for better precision. The Hounsfield Unit (HU) [23] value was adjusted for the 3D modeling.

(1) Cranial 3D modeling

The HU values for the skull were set within the designated average range in Mimics software (Mimics, version 22.0, Materialise, Leuven, Belgium), Min 500 HU and Max 3071 HU, for the masking process. Additionally, unnecessary noise, soft tissues, and bones were removed using the ‘Edit mask’ function. After removal, the completed data were converted to STL files using the ‘CalCulate Part’ function. Subsequently, for precise measurements, ‘points’ were created in the ‘Analyze’ section for each measurement item, and measurements were conducted for each item using the ‘Distance’ function.

(2) Nasal cavity 3D modeling

Since there is no predefined average range for the HU values of the nasal cavity in Mimics software, masking was conducted in custom mode. Initially, a new mask was created with settings at Min −1024 HU and Max −265 HU, and then to exclusively extract the nasal cavity, the ‘Crop Mask’ feature was used to set the region containing the nasal cavity in three views: coronal, sagittal, and frontal. Subsequently, the ‘Edit mask’ feature was used to remove noise. The completed data were converted to STL files using the ‘CalCulate Part’ function. For precise measurements, ‘points’ were created in the ‘Analyze’ section for each measurement item, and measurements were conducted for each item using the ‘Distance’ function.

(3) Soft tissue 3D modeling

The HU values for soft tissues were set within the designated average range in Mimics software, Min −390 HU and Max 160 HU, for the masking process. Additionally, unnecessary noise and bones were removed using the ‘Edit mask’ function. After removal, the completed data were converted to STL files using the ‘CalCulate Part’ function. For precise measurements, ‘points’ were created in the ‘Analyze’ section for each measurement item, and measurements were conducted for each item using the ‘Distance’ function.

#### 2.2.3. Measurement Parameters

All measurements were taken after calibrating the Frankfort horizontal line for precision. To ensure accuracy, the analyze point feature was used to mark points and measure the distance between the points. The measurements were taken at the highest point. Two measurements were taken by Kim and Lee (one each), and the average values were calculated for a reliability assessment (Cronbach’s α = 0.623) before statistical analysis. Furthermore, FI and NI were computed based on the measurements.

(1) Facial index (FI)

**Figure 1:**
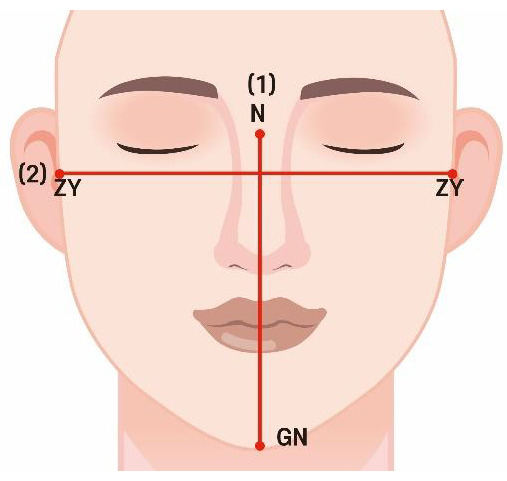
Facial index measurement. (1) FH is the distance between the nasion (n) and gnathion (gn); (2) FW is the bizygomatic width (zygion–zygion).

The FI classifies facial appearance into five types based on facial height (FH) and facial width (FW). FH is the distance between the nasion (n) and gnathion (gn), and FW is the bizygomatic width (zygion–zygion) (Figure 1). Classifications were made using the formula: FH/FW × 100 [28]. Table 1 shows the classification results.

(2) NI

**Figure 2:**
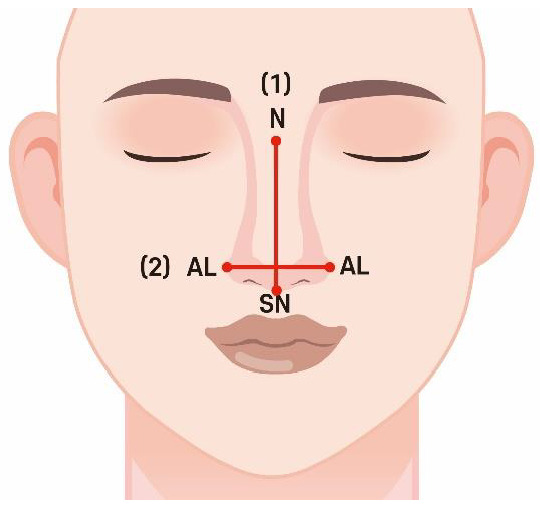
Facial index measurement. (1) NH is the distance between the nasion (n) and subnasale (sn); (2) NW is the distance between the alaria (al).

The NI classifies the nasal appearance into five types based on the nasal width (NW) and nasal height (NH). NW is the distance between the alaria (al), and NH is the distance between the nasion (n) and subnasale (sn) (Figure 2). Classifications were made using the formula: NW/NH × 100 [29]. Table 2 shows the classification results.

(3) Measurement parameters

**Figure 3:**
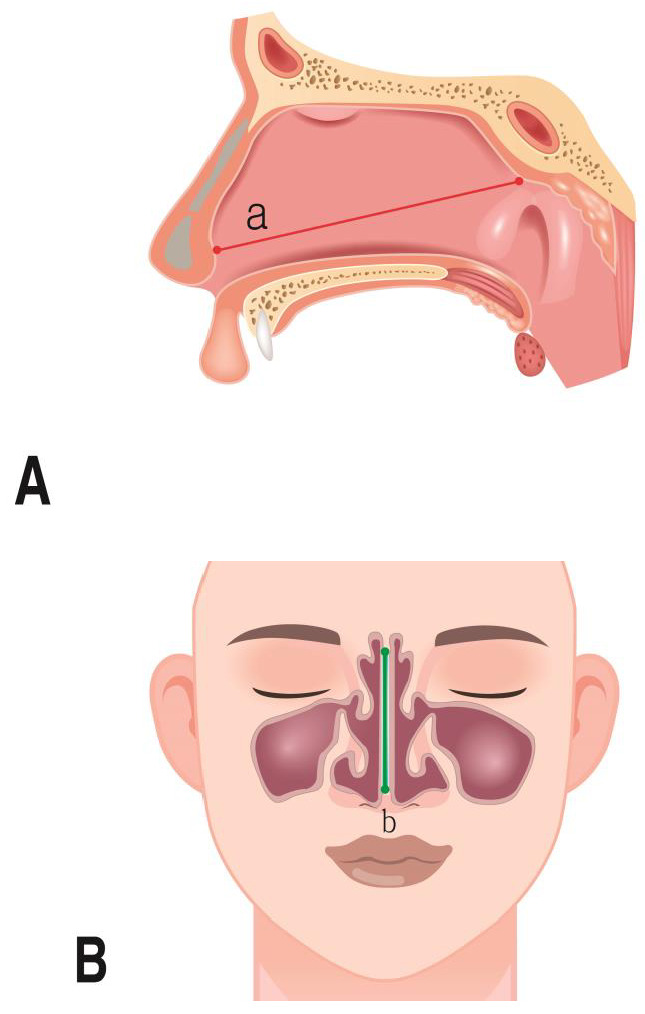
Septum measurement. (**A**) Septum length; a: the distance length of the highest point septum; (**B**) septum height; b: the distance height of the highest point septum.

**Figure 4:**
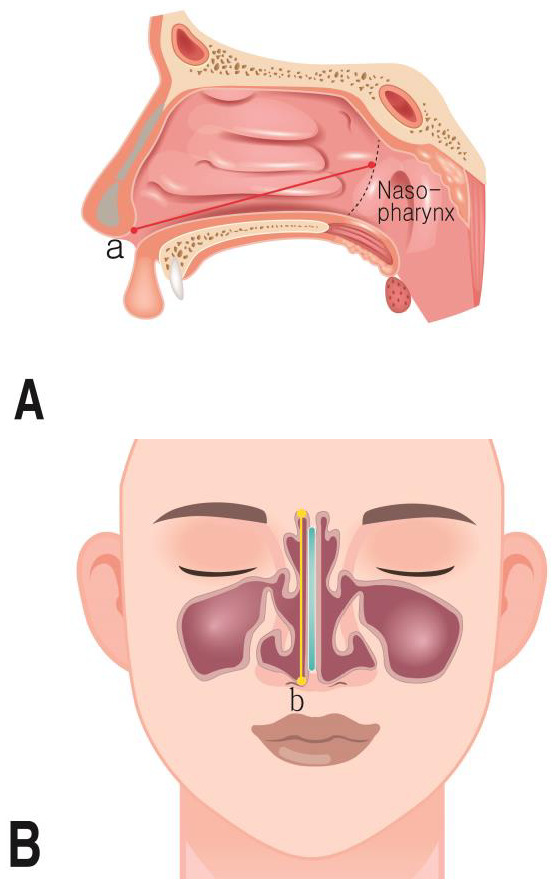
Nasal cavity measurements. (**A**) Nasal cavity length; a: the distance height of the highest point nasal cavities; (**B**) nasal cavity height; b: the distance length of the highest point nasal cavities.

The parameters that were measured for the nasal cavity are shown in Table 3 (Figure 3 and Figure 4).

#### 2.2.4. Three-Dimensional Model Measurement in Mimics Software 

**Figure 5:**
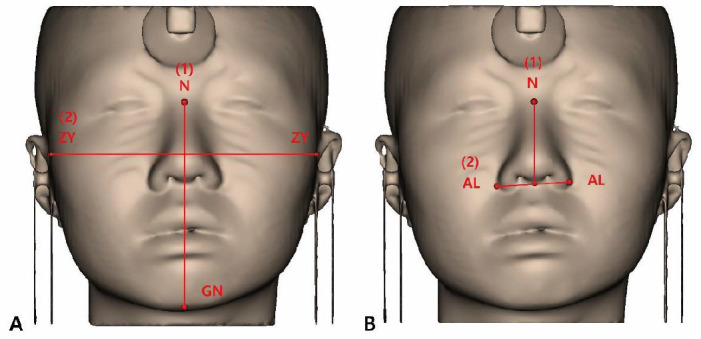
(**A**) Facial index measurement. (1) FH is the distance between the nasion (n) and gnathion (gn); (2) FW is the bizygomatic width (zygion–zygion). (**B**) Facial index measurement. (1) NH is the distance between the nasion (n) and subnasale (sn); (2) NW is the distance between the alaria (al).

**Figure 6:**
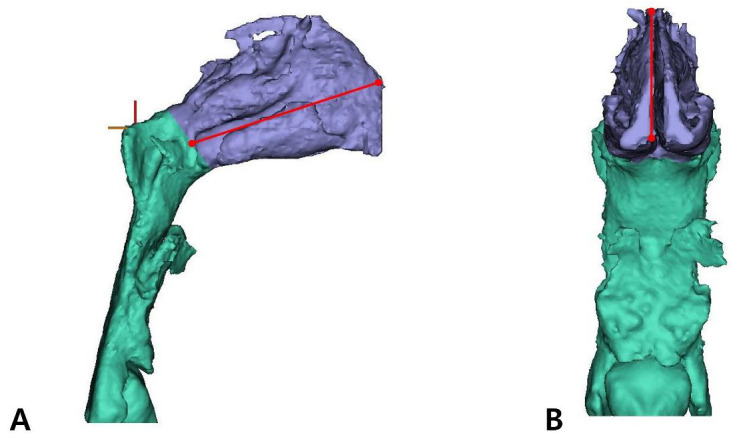
Nasal cavity measurements. (**A**) Nasal cavity length. (**B**) Nasal cavity height.

The method of measuring patient CBCT data after 3D modeling is illustrated in Figure 5 and Figure 6. 

#### 2.2.5. Statistics

Data were analyzed using IBM SPSS Statistics for Windows, version 23 (IBM Corp., Armonk, NY, USA). Differences in nasal cavity sizes according to sex were analyzed using the *t*-test, and differences in nasal cavity size according to FI and NI were analyzed using one-way analysis of variance and the post hoc Scheffe test. For all analyses, 95% confidence intervals (CI) are presented, and the significance level was set at 0.05.

## 3. Results

### 3.1. Nasal Dimensions According to Sex

**Figure 7:**
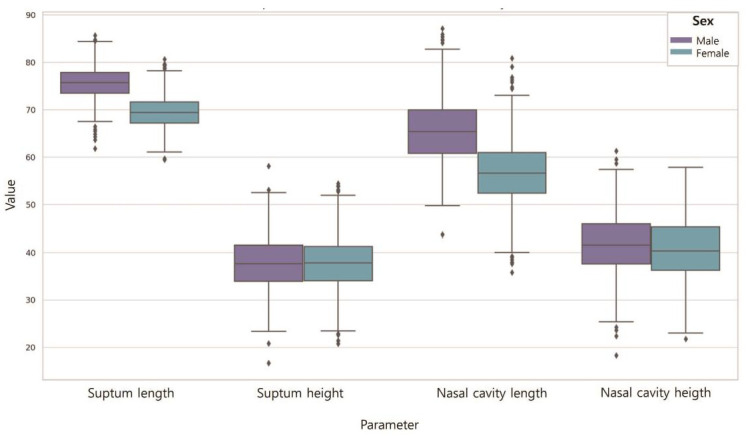
Nasal dimensions according to sex. Septum length was male > female (*p* < 0.001); septum height was male > female (*p* > 0.05). Nasal cavity length was male > female (*p* < 0.001); nasal cavity height was male > female (*p* > 0.05).

Table 4 shows the differences in the nasal cavity sizes according to sex (Figure 7). The septum length of males was longer (75.72) than that of females (69.47) (*p* < 0.001). The septum height of males was higher (37.82) than that of females (37.53). However, the differences were not significant (*p* > 0.05). The length of the nasal cavities of males was higher (65.57) than that of females (56.64) (*p* < 0.001), and the height of the nasal cavities of males was higher (41.82) than that of females (40.51). However, the differences were not significant (*p* > 0.05).

### 3.2. Nasal Dimensions According to the FI

**Figure 8:**
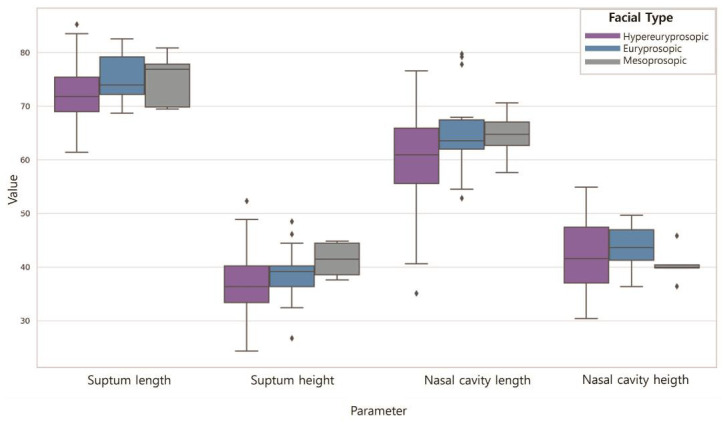
Nasal dimensions according to the FI. Septum length was euryprosopic > mesoprosopic > hypereuryprosopic (*p* < 0.001); septum height was mesoprosopic > euryprosopic > hypereuryprosopic (*p* > 0.05); Nasal cavity length was euryprosopic > mesoprosopic > hypereuryprosopic (*p* < 0.05); nasal cavity height was mesoprosopic > hypereuryprosopic > euryprosopic (*p* > 0.05).

Table 5 shows the differences in the nasal cavity sizes according to the FI (Figure 8). The septum length was longest for those with hypereuryprosopic (71.71), followed by euryprosopic (75.82) and mesoprosopic (72.95) facial shapes (*p* < 0.001). The septum height was highest for those with hypereuryprosopic (37.17), followed by euryprosopic (39.10) and mesoprosopic (39.49) facial shapes; however, the difference was not statistically significant (*p* > 0.05). The length of the nasal cavities was longest for those with hypereuryprosopic (59.74), followed by euryprosopic (65.89) and mesoprosopic (62.39) facial shapes (*p* < 0.05). The height of the nasal cavities was highest for those with hypereuryprosopic (41.05), followed by euryprosopic (40.79) and mesoprosopic (44.39) facial shapes; however, the difference was not statistically significant (*p* > 0.05).

### 3.3. Nasal Dimensions According to the NI

**Figure 9:**
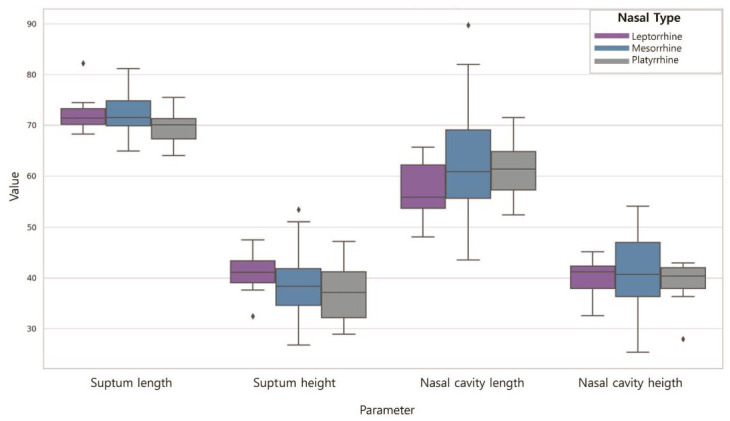
Nasal dimensions according to the NI. Septum length was mesorrhine > leptorrhine > platyrrhine (*p* > 0.05); septum height was leptorrhine > mesorrhine > platyrrhine (*p* > 0.05); nasal cavity length was leptorrhine > mesorrhine > platyrrhine (*p* > 0.05); nasal cavity height was mesorrhine > leptorrhine > platyrrhine (*p* > 0.05).

Table 6 shows the differences in the nasal cavity sizes according to the NI (Figure 9). The septum length was longest for those with leptorrhine (72.28), followed by mesorrhine (73.02) and platyrrhine (70.53) nose shapes. The septum height was highest for those with leptorrhine (39.93), followed by mesorrhine (37.46) and platyrrhine (37.22) nose shapes. The length of the nasal cavities was longest for those with leptorrhine (61.53), followed by mesorrhine (61.32) and platyrrhine (59.63) nose shapes. The length of the nasal cavities was highest for those with leptorrhine (39.71), followed by mesorrhine (41.83) and platyrrhine (38.60) nose shapes. However, the differences were not significant for any of them (*p* > 0.05).

## 4. Discussion

The shape of the nasal cavity holds significant value not only in clinical contexts but also in forensic science [30]. However, the majority of morphometric studies have primarily focused on sex comparisons [1,2,3,4,5,6,7,8,9,10,11,12,13,14,19,20,21,22,23,24,25,26,27,28,29,30,31,32,33,34,35,36,37,38]. Therefore, it is necessary to examine how the nasal dimensions relate to the facial and nose dimensions. In this study, we aimed to anthropometrically compare nasal cavities based on sex, FI, and NI. FI and NI classifications were performed using 3D modeling.

The following results were obtained for the differences in nasal cavity size according to sex. The values of the septum length and height, and nasal cavity length and height of males were higher than that of females on average, similar to previous study findings [31,32]. Samoliński et al. [33] reported that the nasal cavity size varies across ages, sexes, and races. In addition, LoMauro et al. [34] reported that males have larger lungs and longer chest walls and diaphragms than females. Hence, males seem to have larger nasal cavities, which are influenced by the environment and climate. Thus, further research on sex-specific respiration and special considerations in clinical practice must be conducted. 

The following results were obtained for the comparison of nasal cavity sizes according to the FI. Individuals with a broader or “euryprosopic” facial type had longer septum lengths, while those with a rounder or “mesoprosopic” facial type had higher septum heights. Additionally, the length of the nasal cavity was greater in individuals with a broader, euryprosopic facial type, while the height was greater in those with a rounder, mesoprosopic facial type. Thus, euryprosopic types generally tend to have a wider dimension, whereas mesoprosopic types tend to have taller dimensions. According to Butaric et al. [35], there may be potential variations in nasal cavity due to skull size and ecological characteristics. Hence, we were able to confirm that the facial skeletal structure influences the nasal cavity, highlighting the need for special considerations in clinical practice for nose-related care and research in forensic medicine.

The following results were obtained for the comparison of nasal cavity sizes according to the NI. The septum length was greater in the typical mesorrhine type, while height was greater in the elongated and narrow leptorrhine type. The length of the nasal cavity was greater in the elongated and narrow leptorrhine type, while height was greater in the typical mesorrhine type. Tomkinson et al. [36] compared the nasal cavity according to individual characteristics, such as height, weight, and facial width, and reported that the alar width is correlated with the size of the nasal cavity. We also observed that the nasal cavity size differs according to the nasal shape. Furthermore, Leong et al. [37] reported that the external proportions of the nose may be altered by physiological processes involving the internal structures. Therefore, nasal proportions should be considered when conducting nose-related research and clinical surgeries.

This study has the following limitations. Although there were differences in the mean measurements of nasal cavity sizes according to sex, FI, and NI, the differences were not statistically significant. Maddux et al. [38] reported that the inner structure of the nasal cavity is dependent on climate. Further, the nose is longer and larger relative to the cranium in cold and dry environments than that in hot and humid environments. This may be the reason underlying the lack of significant results in the differences in nasal cavity size according to sex, FI, and NI. Therefore, further research is needed to investigate nasal cavity sizes in relation to various factors and dimensions other than sex and skeletal dimensions, in consideration of the climate and environment. 

In this study, we leveraged 3D modeling techniques to observe differences in nasal cavity sizes in relation to sex, FI, and NI. These findings are interpreted as indicative of the relationship between the structure of the nose and facial morphology and are deemed to hold significant implications for future research in the fields of forensic science and clinical practice. In the clinical domain, understanding the variations in nasal cavity sizes according to FI and NI indices could facilitate clinical diagnoses and surgical procedures. Specifically, this knowledge can be instrumental in planning surgeries for conditions requiring personalized treatment plans, such as nasal obstruction and obstructive sleep apnea, by considering the structure of the nose and individual anatomical differences. Consequently, this can aid in surgical outcomes for rhinoplasty and facial reconstruction surgeries, ultimately minimizing post-surgical complications, enhancing respiratory function and aesthetic results, and improving patient satisfaction and quality of life.

The results of this study have illustrated variability among groups by comparing nasal cavity sizes across genders, as well as FI and NI indices. This variability can be utilized to refine methods used in forensic anthropology for skull analysis, providing a more nuanced approach to the identification process. For instance, measurements of the nasal cavity dimensions could be associated with specific population groups when unidentified human remains are discovered, significantly improving the success rate of personal identification. Therefore, the outcomes derived from the anthropometric assessment using 3D modeling in this study are expected to benefit forensic practitioners in their tasks.

## 5. Conclusions

The findings of this study showed that nasal cavity sizes generally differed according to sex, FI, and NI. These results are expected to establish a crucial basis for the enhancement of tailored approaches within the realms of forensic and clinical medicine. Furthermore, this investigation has elucidated the substantial impact that anatomical variations within the nasal cavity may hold in both clinical and forensic settings, thereby underscoring the necessity for additional exploratory efforts concerning the nasal cavity. Importantly, it is anticipated that our findings will serve as foundational data for subsequent studies examining how the structural attributes of the nasal cavity are modulated by external environmental factors, such as the climate.

## Figures and Tables

**Table 1:** Facial index classification.

Facial Index	Range of FI	N
Hypereuryprosopic (very broad face)	80%	75
Euryprosopic (broad face)	80~85%	20
Mesoprosopic (round face)	85~90%	5

**Table 2:** Nasal index classification.

Nasal Index	Range of FI	N
Leptorrhine (long and narrow)	55~69.9%	10
Mesorrhine (moderate shape)	70~84.9%	76
Platyrrhine (broad and short)	85~99.9%	14

**Table 3:** Nasal cavity measurement parameters.

Parameter	Definition
Septum length	Width of the nasal septum in sagittal view
Septum height	Height of nasal septum in coronal view
Nasal cavity width	Width of nasal cavities in coronal view
Nasal cavity height	Height of nasal cavities in coronal view
Nasal cavity length	Width of nasal cavities in sagittal view

**Table 4:** Nasal dimensions according to sex.

Parameter		N	Mean (SD)	F	T	*p*
Septum length	Male	50	75.72 (3.53)	0.085	9.278	0.000 *
	Female	50	69.47 (3.20)			
Septum height	Male	50	37.82 (5.48)	0.000	0.264	0.793
	Female	50	37.53 (5.46)			
Nasal cavity length	Male	50	65.57 (6.46)	0.001	6.864	0.000 *
	Female	50	56.64 (6.56)			
Nasal cavity height	Male	50	41.82 (6.15)	0.275	1.031	0.305
	Female	50	40.51 (6.53)			

Data are mean (standard-deviation values); *p*-values were obtained using *t*-tests (* *p* < 0.001).

**Table 5:** Nasal dimensions according to the FI.

Parameter		N	Mean (SD)	F	*p*
Septum length	Hypereuryprosopic	75	71.71 (4.47)	7.092	0.001 *
	Euryprosopic	20	75.82 (3.83)		
	Mesoprosopic	5	72.95 (4.14)		
Septum height	Hypereuryprosopic	75	37.17 (5.36)	1.285	0.281
	Euryprosopic	20	39.10 (5.70)		
	Mesoprosopic	5	39.49 (5.51)		
Nasal cavity length	Hypereuryprosopic	75	59.74 (7.53)	5.297	0.007 *
	Euryprosopic	20	65.89 (7.75)		
	Mesoprosopic	5	62.39 (7.25)		
Nasal Cavity height	Hypereuryprosopic	75	41.05 (6.55)	0.688	0.505
	Euryprosopic	20	40.79 (5.03)		
	Mesoprosopic	5	44.39 (8.21)		

Data are mean (standard-deviation values); *p*-values were obtained using one-way ANOVA (* *p* < 0.001).

**Table 6:** Nasal dimensions according to the NI.

Parameter		N	Mean (SD)	F	*p*
Septum length	Leptorrhine	10	72.28 (4.16)	1.792	0.172
	Mesorrhine	76	73.02 (3.77)		
	Platyrrhine	14	70.53 (3.77)		
Septum height	Leptorrhine	10	39.93 (3.95)	0.964	0.385
	Mesorrhine	76	37.46 (5.69)		
	Platyrrhine	14	37.22 (4.93)		
Nasal cavity length	Leptorrhine	10	61.53 (7.77)	0.284	0.753
	Mesorrhine	76	61.32 (8.41)		
	Platyrrhine	14	59.63 (4.56)		
Nasal cavity height	Leptorrhine	10	39.71 (5.56)	1.861	0.161
	Mesorrhine	76	41.83 (6.57)		
	Platyrrhine	14	38.60 (4.96)		

Data are mean (standard-deviation values); *p*-values were obtained using one-way ANOVA (* *p* < 0.001).

## Data Availability

Original data are available upon request to the corresponding author.

## References

[1] Tian L., Dong J., Shang Y., Tu J. (2022). Detailed comparison of anatomy and airflow dynamics in human and cynomolgus monkey nasal cavity. Comput. Biol. Med..

[2] Im S., Heo G.E., Jeon Y.J., Sung H.J., Kim S.K. (2014). Tomographic PIV measurements of flow patterns in a nasal cavity with geometry acquisition. Exp. Fluids.

[3] Luo H., Wang J., Zhang S., Mi C. (2018). The application of frontal sinus index and frontal sinus area in sex estimation based on lateral cephalograms among Han nationality adults in Xinjiang. J. Forensic Leg. Med..

[4] Wilkinson C.M. (2010). Facial reconstruction—Anatomical art or artistic anatomy?. J. Anat..

[5] Hsiao J.H., Cottrell G. (2008). Two fixations suffice in face recognition. Psychol. Sci..

[6] Guyomarc’h P., Stephan C.N. (2012). The validity of ear prediction guidelines used in facial approximation. J. Forensic Sci..

[7] Guyomarc’h P., Dutailly B., Charton J., Santos F., Desbarats P., Coqueugniot H. (2014). Anthropological facial approximation in three dimensions (AFA 3D): Computer-assisted estimation of the facial morphology using geometric morphometrics. J. Forensic Sci..

[8] Stephan C.N. (2003). Facial approximation: An evaluation of mouth-width determination. Am. J. Phys. Anthropol..

[9] Rogers T.L. (2005). Determining the sex of human remains through cranial morphology. J. Forensic Sci..

[10] Williams B.A., Rogers T.L. (2006). Evaluating the accuracy and precision of cranial morphological traits for sex determination. J. Forensic Sci..

[11] Krishan K., Chatterjee P.M., Kanchan T., Kaur S., Baryah N., Singh R.K. (2016). A review of sex estimation techniques during examination of skeletal remains in forensic anthropology casework. Forensic Sci. Int..

[12] Scendoni R., Kelmendi J., Arrais Ribeiro I.L., Cingolani M., De Micco F., Cameriere R. (2023). Anthropometric analysis of orbital and nasal parameters for sexual dimorphism: New anatomical evidences in the field of personal identification through a retrospective observational study. PLoS ONE.

[13] Chen F., Chen Y., Yu Y., Qiang Y., Liu M., Fulton D., Chen T. (2011). Age and sex related measurement of craniofacial soft tissue thickness and nasal profile in the Chinese population. Forensic Sci. Int..

[14] Lee U.Y., Kim H., Song J.K., Kim D.H., Ahn K.J., Kim Y.S. (2020). Assessment of nasal profiles for forensic facial approximation in a modern Korean population of known age and sex. Leg. Med..

[15] Jayakrishnan J.M., Reddy J., Kumar R.V. (2021). Role of forensic odontology and anthropology in the identification of human remains. J. Oral Maxillofac. Pathol..

[16] Akbar A., Chatra L., Shenai P.K., VeenaK M., Prabhu R.V., Shetty P. (2018). Dental age estimation by pulp/tooth volume ratio based on cbct tooth images: A forensic study. Indian J. Med. Res..

[17] Sujatha S., Azmi S.R., Devi B.K., Shwetha V., Kumar T.P. (2017). CBCT-the newfangled in forensic radiology. J. Dent. Orofac. Res..

[18] Denny C., Bhoraskar M., Shaikh S.A.A., Bastian T.S., Sujir N., Natarajan S. (2023). Investigating the link between frontal sinus morphology and craniofacial characteristics with sex: A 3D CBCT study on the South Indian population. F1000Research.

[19] Doni R.P., Janaki C.S., Vijayaraghavan V., Raj U.D. (2013). A study on measurement and correlation of cephalic and facial indices in male of South Indian population. Int. J. Med. Res. Health Sci..

[20] Trivedi H., Azam A., Tandon R., Chandra P., Kulshrestha R., Gupta A. (2017). Correlation between morphological facial index and canine relationship in adults—An anthropometric study. J. Orofac. Sci..

[21] Maalman R.S.E., Abaidoo C.S., Darko N.D., Tetteh J. (2019). Facial types and morphology: A study among Sisaala and Dagaaba adult population in the Upper West Region, Ghana. Sci. Afr..

[22] Porter J.P., Olson K.L. (2003). Analysis of the African American female nose. Plast. Reconstr. Surg..

[23] Mehlum C.S., Rosenberg T., Dyrvig A.K., Groentved A.M., Kjaergaard T., Godballe C. (2018). Can the Ni classification of vessels predict neoplasia? A systematic review and meta-analysis. Laryngoscope.

[24] Alex F.R., Steven B., Timothy G.L. (1996). Human Body Composition.

[25] Olotu J.E., Eroje A., Oladipo G.S., Ezon-Ebidor E. (2009). Anthropometric study of the facial and nasal length of adult Igbo ethnic group in Nigeria. Int. J. Biol. Anthropol..

[26] Esomonu U.G., Ude R.A., Lukpata P.U., Nandi E.M. (2013). Anthropometric study of the nasal index of Bekwara ethnic group of Cross River state, Nigeria. Int. Res. J. Appl. Basic. Sci..

[27] Peter F., Jorgen T.J. (2001). Anatomy in Diagnostic Imaging.

[28] Pandeya A., Atreya A. (2018). Variations in the facial dimensions and face types among the students of a Medical College. JNMA J. Nepal Med. Assoc..

[29] Milanifard M., Hassanzadeha G. (2018). Anthropometric study of nasal index in Hausa ethnic population of northwestern Nigeria. J. Contemp. Med. Sci..

[30] Liu Y., Johnson M.R., Matida E.A., Kherani S., Marsan J. (2009). Creation of a standardized geometry of the human nasal cavity. J. Appl. Physiol..

[31] Russel S.M., Frank-Ito D.O. (2023). Gender differences in nasal anatomy and function among Caucasians. Facial Plast. Surg..

[32] Wang J.J., Chiang Y.F., Jiang R.S. (2023). Influence of Age and Gender on Nasal Airway Patency as Measured by Active Anterior Rhinomanometry and Acoustic Rhinometry. Diagnostics.

[33] Samoliński B.K., Grzanka A., Gotlib T. (2007). Changes in nasal cavity dimensions in children and adults by gender and age. Laryngoscope.

[34] LoMauro A., Aliverti A. (2018). Sex differences in respiratory function. Breathe.

[35] Butaric L.N., McCarthy R.C., Broadfield D.C. (2010). A preliminary 3D computed tomography study of the human maxillary sinus and nasal cavity. Am. J. Biol. Anthropol..

[36] Tomkinson A., Eccles R. (1995). External facial dimensions and minimum nasal cross-sectional area. Clin. Otolaryngol..

[37] Leong S.C., Eccles R. (2009). A systematic review of the nasal index and the significance of the shape and size of the nose in rhinology. Clin. Otolaryngol..

[38] Maddux S.D., Butaric L.N., Yokley T.R., Franciscus R.G. (2017). Ecogeographic variation across morphofunctional units of the humannose. Am. J. Biol. Anthropol..

